# Inflammatory Determinants of Pregravid Obesity in Placenta and Peripheral Blood

**DOI:** 10.3389/fphys.2018.01089

**Published:** 2018-08-07

**Authors:** Suhas Sureshchandra, Nicole E. Marshall, Randall M. Wilson, Tasha Barr, Maham Rais, Jonathan Q. Purnell, Kent L. Thornburg, Ilhem Messaoudi

**Affiliations:** ^1^Department of Molecular Biology and Biochemistry, University of California, Irvine, Irvine, CA, United States; ^2^Maternal-Fetal Medicine, Oregon Health & Science University, Portland, OR, United States; ^3^Division of Biomedical Sciences, School of Medicine, University of California, Riverside, Riverside, CA, United States; ^4^Department of Medicine, The Knight Cardiovascular Institute, Oregon Health & Science University, Portland, OR, United States

**Keywords:** maternal obesity, inflammation, placenta, microbiome, RNA-Seq, PBMC, cytokines and inflammation

## Abstract

Pre-pregnancy (pregravid) obesity has been linked to several adverse health outcomes for both mother and offspring. Complications during pregnancy include increased risk for gestational diabetes, hypertension, preeclampsia, placental abruption, and difficulties during delivery. Several studies suggest that these negative outcomes are mediated by heightened systemic inflammation as well as changes in placental development and function. However, the molecular mechanisms by which pregravid obesity affects these processes are poorly understood. In this study, we aimed to address this question by carrying out a comprehensive analysis of the systemic maternal immune system coupled with placental gene expression and microbial profiling at term delivery (11 lean and 14 obese). Specifically, we examined the impact of pregravid obesity on circulating cytokines, chemokine, adipokines, and growth factors using multiplex Luminex assay. Innate and adaptive immune cell frequencies and their cytokine production in response to stimuli were measured using flow cytometry. Finally, changes in placental transcriptome and microbiome were profiled using RNA- and 16S-sequencing, respectively. Pregravid obesity is characterized by insulin and leptin resistance, high levels of circulating inflammatory markers IL-6 and CRP, in addition to chemokine IL-8 (*p* < 0.01). Moreover, pregravid obesity was associated with lower frequency of naïve CD4+ T-cells (*p* < 0.05), increased frequency of memory CD4+ T-cells (*p* < 0.01), and a shift towards Th2 cytokine production (*p* = 0.05). Myeloid cells from the obese cohort produced higher levels of pro-inflammatory cytokines but lower levels of chemokines following TLR stimulation (*p* < 0.05). Lastly, pregravid obesity is associated with increased abundance of Bacteroides and changes in the expression of genes important for nutrient transport and immunity (FDR < 0.05). Collectively, these data indicate that pregravid obesity is associated with heightened systemic inflammation and of dysregulated nutrient transport in the placenta and provide insight into the basis of fetal reprogramming.

## Introduction

Approximately 37% of women of reproductive age (20–40 years of age) are considered obese (>30 kg/m2) (Flegal et al., 2016). Pre-pregnancy (pregravid) obesity is associated with significant adverse health outcomes for both mother and offspring (Poston et al., 2016). The risks to the mother include gestational hypertension, preeclampsia, gestational diabetes mellitus, placental abruption, and complications during delivery (Gaillard et al., 2013). Fetal complications include neural tube defects, stillbirth, and macrosomia (McMahon et al., 2013; Gaudet et al., 2014). Adverse outcomes for the offspring include more frequent admissions to neonatal intensive care unit (NICU) due to bacterial sepsis and necrotizing enterocolitis (Rastogi et al., 2015; Suk et al., 2016), higher incidence of metabolic disease, allergy/wheezing, cancer, as well as altered neurodevelopment (Godfrey et al., 2017).

The mechanisms underlying these adverse outcomes are poorly understood, but chronic low-grade maternal inflammation has emerged as a key contributor to the pathophysiological changes associated with pregravid obesity (Denison et al., 2010). In support of this view, studies have reported increased plasma levels of insulin and leptin as well as inflammatory mediators such as IL-6 and C-reactive protein (CRP) with pregravid obesity (Schmatz et al., 2010). Additionally, increased macrophage accumulation in both the adipose tissue and the placenta has been observed in obese pregnant women at 37 weeks of gestation (Basu et al., 2011). Furthermore, pregravid obesity is associated with transcriptional changes in genes that regulate inflammation, lipid metabolism, insulin resistance, angiogenesis, and hormone activity in the placenta (Saben et al., 2014a,b).

Nonetheless, our current understanding of the interplay between systemic and placental inflammation with pregravid obesity is limited due to the lack of multi-dimensional studies that simultaneously examine alterations in circulating immune mediators and cells as well as placenta from the same subjects. Moreover, it is still unclear if placental microbiome in term gestation varies with pre-pregnancy BMI. To address these questions, we measured alterations in the frequency of peripheral immune cells, circulating levels of systemic inflammatory and metabolic mediators, placental transcriptome and microbiome to better understand the impact of pregravid obesity on the establishment of an inflammatory environment.

## Materials and Methods

### Subjects

This study was approved by the Institutional Ethics Review Boards of Oregon Health and Science University and the University of California, Irvine. A total of 25 non-smoking women without diabetes who had an uncomplicated, singleton pregnancy were enrolled at 37 weeks of gestation and initially grouped according to pregravid body mass index (BMI): 11 lean women with a mean age of 31.5 ± 4.95 years and pre-pregnancy BMI of 22.27 ± 1.95 kg/m^2^; and 14 obese women with a mean age of 29.6 ± 5.9 years and pre-pregnancy BMI of 37.5 ± 5.0 kg/m^2^. Written informed consent was obtained from all participants. The racial distribution was as follows: 19 Caucasian, 2 Asian-American/Pacific Islander, 1 American-Indian/Alaskan native, 1 African-American, and 2 unknown. Exclusion criteria included active maternal infection, documented fetal congenital anomalies, substance abuse, chronic illness requiring regular medication use, gestational diabetes, chorioamnionitis, significant medical conditions (active cancers, cardiac, renal, hepatic, or pulmonary diseases), or an abnormal glucose tolerance test.

Subjects presented for their study visit at 37–38 weeks of gestation and underwent a fasting blood draw and body composition measurement via air displacement plethysmography by using a BodPod (Life Measurement Inc.). This time point was chosen to avoid confounding factors associated with labor, mode of delivery, or variations in gestational age. Due to the strong positive correlation between pre-pregnancy BMI and total body fat determined using the BodPod approach (*r* = 0.82; *p* < 0.0001) [(Wilson et al., 2015) and **Supplementary Figure 1A**], subjects were stratified as lean or obese based on pre-pregnancy BMI.

Power analysis was performed using GPower software using circulating levels of CRP from an earlier larger study (Caporaso et al., 2011), using differences between two independent means. With these reported values, the minimum number of samples to detect comparable differences in CRP with a power of 0.99 (type I error = 0.05) is *n* = 9.

### Plasma and Peripheral Blood Mononuclear Cell (PBMC) Isolation

Complete blood counts were obtained by Beckman Coulter Hematology analyzer (Brea, CA, United States). Peripheral blood mononuclear cells (PBMC) and plasma were obtained by standard density gradient centrifugation over the blood separation polymer Ficoll (GE Healthcare Life Sciences, Pittsburg, PA, United States). PBMC were frozen in 10% DMSO/FBS and stored in liquid nitrogen while plasma was stored at -80°C until analysis.

### Cytokines, Chemokines, and Growth Factor Measurements

Levels of immune mediators in plasma and supernatants were measured using a human cytokine/chemokine/growth factor pre-mixed 38-plex panel (eBioscience, San Diego, CA, United States) and an 8-plex human adipokine kit (Millipore, Temecula, CA, United States). Samples were analyzed using a MAGPIX Instrument (Luminex, Austin, TX, United States). CRP and IL-6 were measured using a high sensitivity ELISA (Life Technologies, Carlsbad, CA, United States).

### Endotoxin Measurements

Plasma LPS levels were measured using Limulus Amoebocyte Lysate (LAL) assay (Hycult Biotech, Uden, Netherlands) on undiluted samples according to the manufacturer’s instructions.

### Flow Cytometry

We analyzed frequencies of immune cell subsets within PBMC (1–2 × 10^6^ cells) using the staining panel we previously described (Sureshchandra et al., 2017). Briefly, 1–2 × 10^6^ PBMC were stained using antibodies against: CD4 (OKT4), CD8b (2ST8.5H7), CD95 (Dx2), CD28 (28.2), CCR7 (G043H7), CD20 (2H7), CD27 (O323), and IgD (C4211) to delineate naïve and memory T and B cells populations. A second tube of PBMC was stained using antibodies against: CD3 (UCHT1), CD20 (2H7), HLA-DR (LN3), CD14 (M5E2), CD11c (3.9), CD123 (6H6,), CD56 (RPA-T8), and CD16 (3G8) to delineate monocytes, myeloid dendritic cells (mDC); plasmacytoid dendritic cells (pDC), and natural killer (NK) cell subsets, respectively. All flow cytometry samples were acquired with LSRII instrument (Becton Dickinson, Franklin Lakes, NJ, United States) and analyzed using FlowJo (TreeStar, Ashland, OR, United States).

### T Cell Stimulation Assay

1–2 × 10^6^ PBMC were stimulated for 24 h at 37°C in RPMI supplemented with 10% FBS in the presence or absence of anti-CD3/CD28 (OKT3; CD28.2); Brefeldin A (Sigma, St. Louis, MO, United States) was added after 1 h incubation. Cells were stained for CD4 and CD8, fixed, permeabilized, and stained intracellularly for TNFα (MAb11), IFNγ (4S.B3), IL-4 (8D4-8), IL-2 (MQ1-17H12), and IL-17α (BL168) as previously described (Sureshchandra et al., 2017).

### Toll-Like Receptor Stimulation Assay

7 × 10^5^ PBMC were cultured for 16 h at 37°C in the absence/presence of either 1 μg/mL Pam3CSK4 (TLR1/2), 1 μg/mL Poly I:C (TLR3), 1 μg/mL LPS (TLR4), 1 μg/mL FSL-1 (TLR2/6), 1 μg/mL Imiquimod (TLR7), or 5 μg/mL ODN2216 (TLR9 agonist) (InvivoGen, San Diego, CA, United States). Production of immune mediators in the supernatants was assessed using a human 45-plex (Thermo Fisher Scientific, Waltham, MA, United States). Samples were analyzed using a MAGPIX Instrument (Luminex, Austin, TX, United States).

### Statistical Analyses

Data sets were first assessed for normality using Shapiro-Wilk test and equality of variances using Levene test. Group differences between datasets normally distributed were tested using unpaired *t*-test (for datasets with equal variances) or unpaired *t*-test with Welch’s correction (for cases with unequal variances). Datasets not normally distributed were subjected to non-parametric testing using Mann-Whitney test. Relationships between BMI and circulating factors were tested using a simple linear regression. Pair-wise relationships between analytes were tested using Pearson’s correlation analysis. The accepted *p*-value for all comparisons was <0.05.

### Placenta DNA Extraction and 16S rRNA Gene Library Preparation

Total DNA was extracted from placental tissue (*n* = 7–8/group) using PowerSoil DNA Isolation Kit (MO BIO Laboratories, Carlsbad, CA, United States). PCR and custom primers were used to amplify the V4–V5 region of the 16S rRNA gene as previously described (Marshall et al., 2016), and sequenced on the Illumina MiSeq platform.

### Analysis of 16S rRNA Data

Low quality and chimeric sequences were removed using Quantitative Insights Into Microbial Ecology (QIIME). Reference operational taxonomic units (OTU) were selected at 97% similarity and taxonomic assignments were made using the 2013 Greengenes reference database (version 13.5). Changes in bacterial abundance at the L7 level was measured using edgeR. False discovery rate (FDR) was controlled at 5%. PICRUSt v1.0.0 was used to predict abundances of Clusters of Orthologous Groups (COGs) from the OTU abundances. Predicted differences in bacterial gene families between lean and obese groups were compared using Bray-Curtis distance. The 16S rRNA gene sequence data have been deposited in NCBI’s Sequence Read Archive (SRA Accession number pending).

### Placenta RNA Isolation and Library Preparation

Total RNA was isolated from placental tissue (*n* = 6–7/group) using the Qiagen mRNAeasy kit (Qiagen, Valencia, CA, United States). RNA concentration and integrity was verified using Agilent 2100 Bioanalyzer. Total RNA was depleted of ribosomal fraction using Ribo-Gone rRNA removal kit. Libraries were then constructed using SMARTer Stranded RNA-Seq Kit (Clontech, Mountain View, CA, United States). Following QC for size, quality and concentrations, libraries were multiplexed and subject to sequencing (75 bp single end) on the NextSeq platform (Illumina, San Diego, CA, United States).

### Analysis of RNA-Seq Data

Quality control of raw reads was performed retaining bases with quality scores of ≥20 and reads ≥50 base pair long. Reads were aligned to human genome (hg38) using splice aware aligner TopHat as previously described (Doria et al., 2010) using annotations available from Ensembl (GRCh38.85) database. Quantification and statistical validation of differentially expressed genes (DEGs) was performed using edgeR, with candidate genes defined by at least two-fold change in expression with multiple hypothesis corrected (Benjamini-Hochberg procedure) FDR of at least 0.05. Functional enrichment of DEGs was performed using open source functional enrichment tool DAVID^1^ and disease associations analyzed using MetaCore^TM^. Heatmaps were generated in R. Gene expression and 16S data have been deposited in NCBI’s Sequence Read Archive BioProject PRJNA478464.

### Data Integration and Association Testing

Association between circulating mediators and placenta gene expression was determined using sparse Partial Least squares (sPLS) regression as described and implemented in R package mixOmics. Correlations between placental gene expression and BMI were calculated using R.

## Results

### Pregravid Obesity Alters Blood Levels of Circulating Factors

**FIGURE 1:**
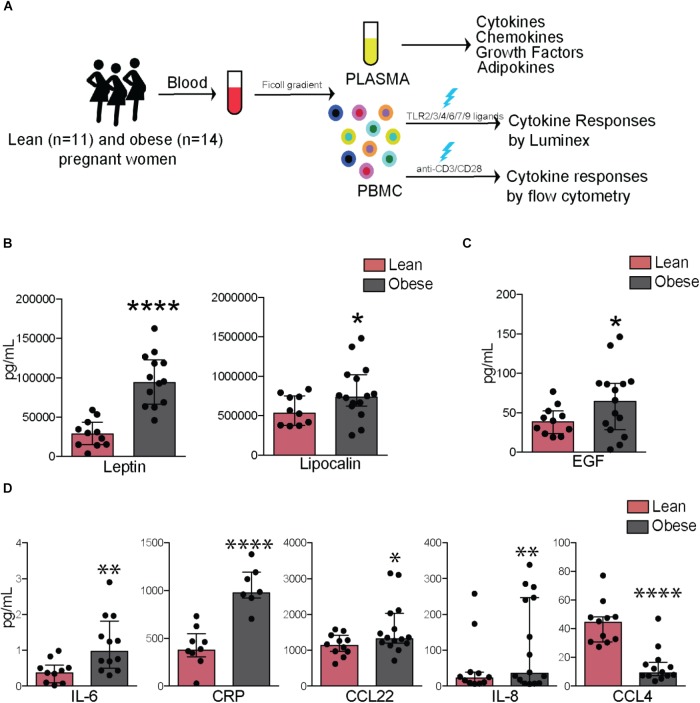
Pregravid obesity alters plasma levels of inflammatory mediators. **(A)** Study design: circulating factors in plasma were measured using Luminex and ELISA while frequency and cytokine production by immune cell subsets were quantified using flow cytometry. **(B)** Plasma levels of leptin and lipocalin 2. **(C)** Plasma levels of EGF. **(D)** Circulating levels of canonical markers of inflammation-IL6 and CRP as well as chemokines CCL22, IL8, and CCL4. Significant differences between groups are indicated as follows: ^∗^*p* < 0.05, ^∗∗^*p* < 0.01, ^∗∗∗^*p* < 0.001, and ^∗∗∗∗^*p* < 0.0001. All of the bar graphs show median values with interquartile ranges (IQR).

Blood samples were collected at 37–38 weeks of gestation in order to determine the impact of pregravid obesity on circulating hormonal and immune factors as well as immune cell frequency and function. Subjects were stratified as lean or obese based on their pre-pregnancy BMI (**Figure 1A**), which showed a strong positive correlation with total body fat mass (*r* = 0.82; *p* < 0.0001) [(Wilson et al., 2015) and **Supplementary Figure 1A**]. We observed no significant differences in mode of delivery (vaginal vs. C-section), gestational age, offspring birth weight, and placenta weight while the ponderal index of the babies born to obese mother was slightly higher than that of babies born to lean mothers (*p* = 0.051) (**Table 1**).

**Table 1:** Pregnancy and neonatal outcomes.

	Lean *N* = 11	Obese *N* = 14	*p*-value
Gestational age at delivery (weeks)	40.2 ± 1.1	40.1 ± 1.1	0.71
Mode of delivery *N* (%)			0.97
Vaginal	8 (72.7)	10 (71.4)	
Primary cesarean	2 (18.2)	3 (21.4)	
Repeat cesarean	1 (9.1)	1 (7.1)	
Birth weight (kg)	3.45 ± 0.49	3.58 ± 0.53	0.54
Infant sex (female)	5 (45.5)	6 (42.9)	0.90
Ponderal index (g/cm^3^)	24.4 ± 1.2	26.7 ± 3.6	0.05
Placental weight (g)	456 ± 86	482 ± 117	0.54

Plasma levels of leptin (*p* < 0.0001) and insulin (*p* < 0.05) varied linearly with BMI (**Supplementary Figure 1B**) and with each other (**Supplementary Figure 1C**). Plasma levels of both leptin (*p* < 0.0001) and lipocalin-2 (*p* < 0.05) were significantly higher in obese subjects (**Figure 1B**), no significant differences or correlations were observed for other adipokines measured (resistin, adipsin, or adiponectin), but significant associations were observed between levels of lipocalin and resistin (*p* < 0.0001) as well as between adipsin and adiponectin (*p* < 0.001) (**Supplementary Figure 1C**). Growth factor EGF was significantly elevated in obese subjects (*p* < 0.05) (**Figure 1C**). In terms of cytokines, plasma IL-6 (*p* < 0.01) and CRP (*p* < 0.0001) were significantly elevated with pregravid obesity (**Figure 1D**). Obese subjects also had elevated levels of the chemokines IL-8 (*p* < 0.01) and CCL22 (*p* < 0.05), but lower levels of CCL4 (*p* < 0.0001) (**Figure 1D**). While circulating levels of growth factors GMCSF and FGF2 did not vary significantly between the two groups, they positively correlated with BMI (*p* = 0.04 and 0.01, respectively; **Supplementary Figure 1D**).

### Pregravid Obesity Is Associated With Altered Cytokine Production and Frequency of CD4+ T-Cell Subsets

**FIGURE 2:**
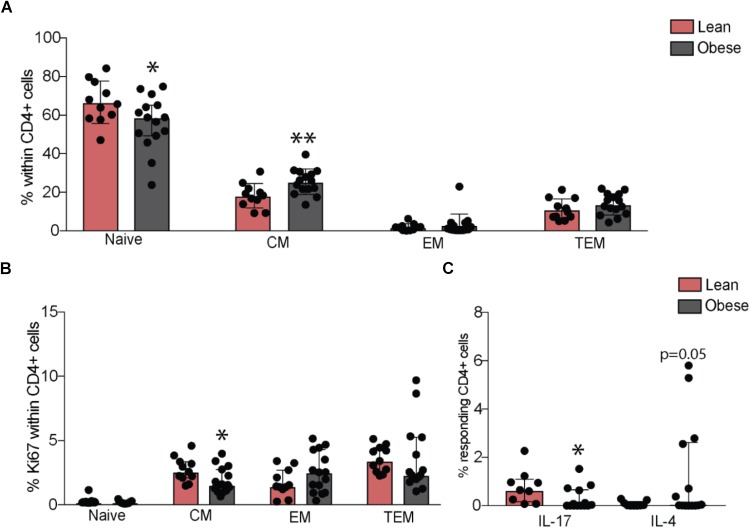
Pregravid obesity alters frequencies of circulating CD4+ T-cells and their responses to stimulation. **(A)** Changes in percentages of circulating naïve and memory CD4+ T-cells subsets measured by flow cytometry and **(B)** their homeostatic proliferation. **(C)** Changes in frequency of IL17 and IL4 secreting CD4+ T-cells following *in vitro* CD3/CD28 activation. Significant differences between groups are indicated as follows: ^∗^*p* < 0.05, ^∗∗^*p* < 0.01, ^∗∗∗^*p* < 0.001, and ^∗∗∗∗^*p* < 0.0001. All of the bar graphs show median values with interquartile ranges (IQR).

Although we observed no differences in the numbers of total lymphocytes or granulocyte populations (**Supplementary Figure 2A**), our analysis revealed a small increase in the frequency of total CD8+ T-cells (*p* = 0.05) in the obese group, in the absence of differences in naïve and memory subsets (**Supplementary Figures 2B,C**). No differences in frequency of total or naïve and memory B cell subsets were observed (**Supplementary Figures 2B,D**). Although total CD4+ T-cell numbers were comparable between the two groups (**Supplementary Figure 2B**), pregravid obesity was associated with lower numbers of naïve and increased numbers of central memory cells (**Figure 2A**), that was accompanied by decreased homeostatic proliferation within the central memory subset (**Figure 2B**).

We next tested changes in T cell function by measuring production of canonical cytokines following *ex vivo* stimulation by activation of CD3/CD28. Obese subjects had reduced frequency of IL-17 secreting (*p* < 0.05) and increased frequency of IL-4 secreting CD4+ T-cells (*p* = 0.05) (**Figure 2C**), suggesting a bias towards Th2 and a suppression of Th17 responses. No differences were observed in frequencies of IL-2, TNFα, or IFNγ-secreting CD4+ T-cells. CD8+ T-cell responses did not differ significantly between the lean and obese subjects post stimulation (**Supplementary Figure 2E**).

### Pregravid Obesity Is Associated With Altered Responses to TLR Activation

**FIGURE 3:**
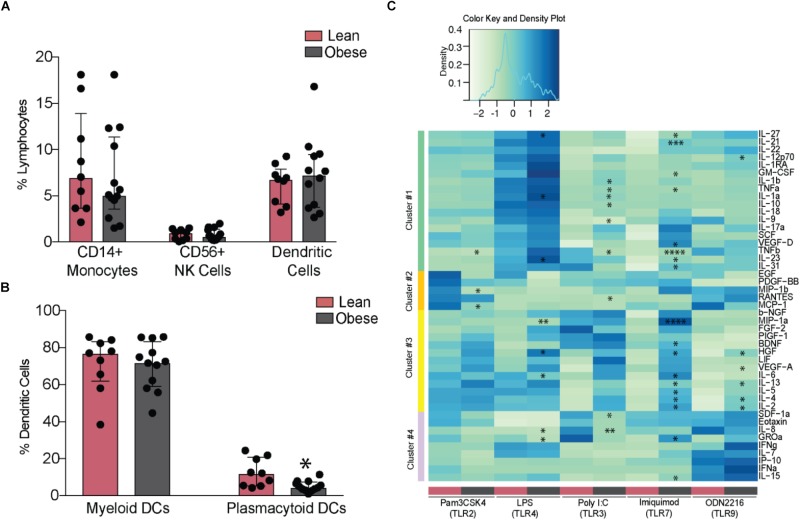
Maternal obesity establishes a hyper-responsive phenotype in circulating monocytes. **(A)** Changes in the frequency of circulating CD14+ monocytes, CD56+ NK cells, and DCs **(B)** Relative frequencies of myeloid and plasmacytoid DCs. **(C)** Clustered heatmap of cytokine, chemokine, and growth factor responses following stimulation with TLR2 (Pam3CSK4), 3 (PolyI:C), 4 (LPS), 7 (Imiquimod), and 9 (ODN2216) agonists. Responses are reported as scaled post stimulatory concentrations in pg/mL. Significant differences between groups are indicated as follows: ^∗^*p* < 0.05, ^∗∗^*p* < 0.01, ^∗∗∗^*p* < 0.001, and ^∗∗∗∗^*p* < 0.0001. All of the bar graphs show median values with inter quartile ranges (IQR).

Analysis of innate immune cells revealed no differences in frequencies of circulating monocytes, NK cells (**Figure 3A**), or their subsets (**Supplementary Figures 3A,B**). Although the frequencies of circulating DCs were similar between the two groups, we detected a marked reduction in pDCs (**Figure 3B**). To test the impact of pregravid obesity on the functional responses of innate immune cells, we measured cytokine, chemokine, and growth factor production from PBMC following overnight stimulation with TLR2 (Pam3CSK4), 3 (PolyI:C), 4 (LPS), 6 (FSL-1), 7 (Imiquimod), and 9 (ODN2216) ligands. Interestingly, spontaneous (in the absence of stimulation) production of several chemokines (IL-8, CXCL10, Eotaxin, CCL4, and CCL2) was significantly reduced with pregravid obesity (**Supplementary Figure 3C**). Similarly, secretion of chemokines following LPS (MIP1a, GROa, and IL-8) and Pam3CSK4 (SDF1a, Eotaxin, MIP1b, and MCP1) stimulation (**Figure 3C** – clusters two and four) was reduced in the obese group. Production of pro-inflammatory mediators (e.g., IL-6, TNFα, IL-1α, and IL-12) was significantly higher in obese subjects, after LPS, PolyI:C, Imiquimod, and ODN2216 stimulation (**Figure 3C** – clusters one and three). Higher levels of regulatory cytokines (IL-4 and IL-13) and growth factors (VEGF, GMCSF, and HGF) (Clusters one and three) were also detected in the obese group following stimulation with PolyI:C (TLR3 ligand), Imiquimod (TLR7 ligand), and ODN2216 (TLR9 ligand). No differences were observed following FSL-1 stimulation (TLR2/TLR6 ligand, data not shown).

### Pregravid Obesity Establishes a Distinct Transcriptome in the Placenta

**FIGURE 4:**
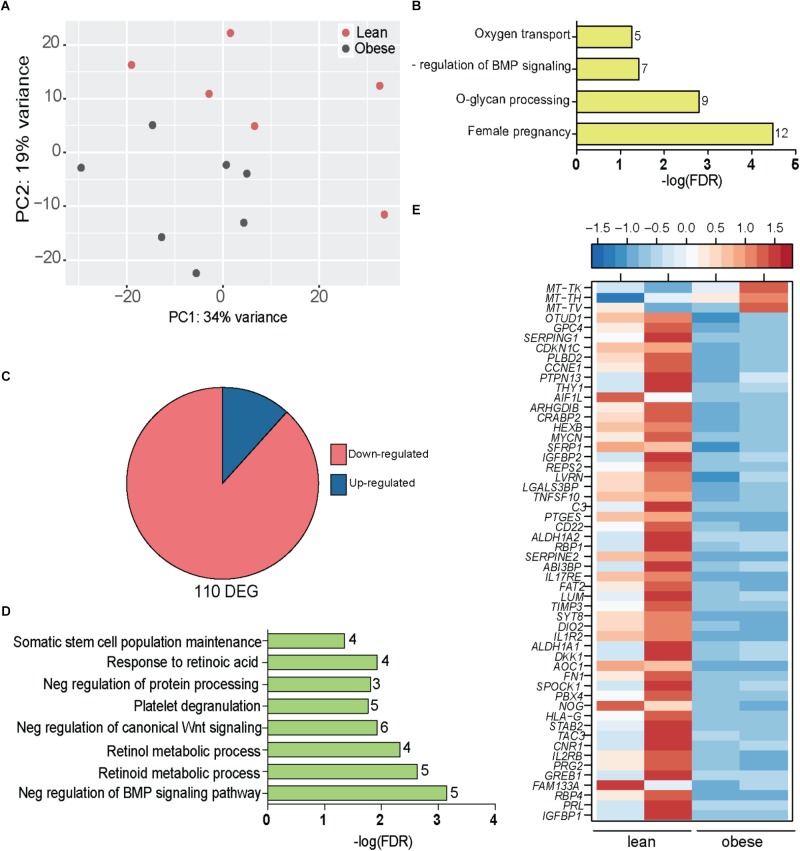
Obesity during pregnancy alters the placental transcriptome. **(A)** Principal Component Analysis of absolute gene expression of 500 most variable genes. **(B)** Functional enrichment of the 500 variable genes evaluated using DAVID. Numbers adjacent to the bar represent number of genes mapping to the gene ontology (GO) term. **(C)** Pie chart denoting number of differentially expressed genes (DEG) identified by edgeR with at least two-fold change in expression and FDR controlled at 5%. **(D)** Functional enrichment of 110 DEG using DAVID. Numbers adjacent to the bar represent number of DEGs mapping to the GO term (FDR < 5%). **(E)** Heatmap of genes mapping to disease terms “Immune System Diseases” and “Pregnancy Complications (FDR < 5%) (See **Supplementary Figure 4A**).

Next, we interrogated changes in gene expression in placentas of lean and obese mothers using RNA-Seq. The most variable genes that separated the placental transcriptional profiles of lean and obese mothers (**Figure 4A**) enriched to gene ontology (GO) terms: “female pregnancy” (*LEP*, *RPL*, *TAC3*, *TGFB3*, *FOS*, *FOSB*, *CRH*, *EPYC*, *PSG4*, *PSG7*, *IGFBP2*, and *HPGD*); “O-glycan processing” (*B3GNT7, GCNT1, MUC12, MUC20, MUC3A, MUC4, MUC5AC, MUC5B*, and *MUC6*); “oxygen transport” (*HBA1*, *HBA2*, *HBB*, *HBG21*, and *HBG2*); and “regulation of Bone Morphogenetic Protein” (BMP) signaling (*CHRDL1*, *DKK1*, *FSTL3*, *NOG*, *SFRP1*, and *WNT5A*) (**Figure 4B**).

Our analysis indentified 110 DEGs that were mostly repressed with pregravid obesity (**Figure 4C**) and significantly enriched to metabolic processes and signaling pathways (**Figure 4D**). In particular, pregravid obesity was associated with suppression of genes involved in retinoid, retinol, and retinoic acid metabolism (*GPC4*, *ALDH1A1*, *ALDH1A2*, *CRABP2, RBP1*, *RBP4*, *SDC4*, and *PTGES*) (**Figure 4D**) as well as developmental pathways, such as BMP (*NOG*, *CHRDL1*, *DKK1*, and *SRFP1*) and Wnt signaling (*NOTUM*, *IGFBP1*, and *IGFBP2*) (**Figure 4D**).

Additional analyses using the Diseases Database available in MetaCore revealed enrichment to the term “pregnancy complications” (**Supplementary Figure 4A**), including genes, such as: *CDKN1C*, an imprinted gene dysregulated in miscarriages or fetal deaths (Bermingham et al., 2000); insulin like growth factors binding proteins *IGFBP1* and *IGFBP1*; and *ASCL2*, a critical regulator of trophoblast development (**Figure 4E**). Additionally, this list included several genes associated with preeclampsia (PE) such as *TIMP3*, Wnt regulator *DKK1*, fibronectin *FN1*, cannabinoid receptor *CNR1*, neurokinin receptor *TAC3*, retinol binding protein *RBP4*, and prolactin *PRL* (**Figure 4E**). DEGs that enriched to “immune system diseases” played critical roles in cell-matrix communication (*THY1*, *LVRN*, *LUM*, *TIMP3*, *SPOCK1*, *LGALS3BP*, *FAT2*, and *SERPINE2*) as well as inflammation (*IL1R2*, *CD22*, *PTGES*, *TNFSF10*, *IL2RB*, *C3*, *SERPING1*, and *IL-17RE*) (**Figure 4E**). Interestingly, *HLA-G*, a gene with central role in both susceptibility to and pathogenesis of PE (O’Brien et al., 2000; Hunt et al., 2006) as well as immune tolerance during pregnancy (Stamilio and Scifres, 2014), was severely downregulated in placentas of obese mothers (**Figure 4E**).

Genes suppressed with pregravid obesity exhibited strong positive associations with circulating levels of GM-CSF and MCP1 and negative associations with PAI-1 and Adipsin (**Supplementary Figure 4B**). This included genes involved in glycosaminoglycan biosynthetic process (*EXTL3*, *GPC4*, and *SDC4*) and cellular response to redox state (*ARHGDIB* and *VASN*). Interestingly, expression of *LVRN*, a metalloprotease that regulates activities of key peptides during placentation, negatively correlated with BMI. The up-regulated genes encoded mitochondrial tRNA genes that strongly correlated with circulating growth factors (VEGF, EGF, and FGF2), adipokines (resistin and leptin), and pro-inflammatory mediators (GM-CSF, IFNγ, and IFNα).

### Maternal Pregravid BMI Is Associated With Limited Changes in Microbial Composition

**FIGURE 5:**
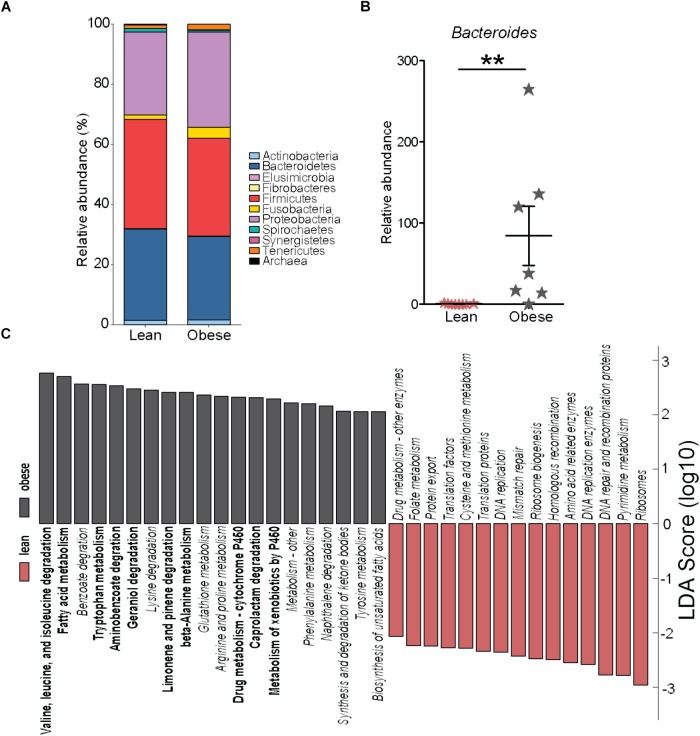
Pregravid obesity is not associated with structural changes in its placental microbiome. **(A)** Median relative abundance of microbial communities at the phylum level reported by QIIME. No significant differences were observed. **(B)** Differences in the relative abundance of Bacteroides detected by both edgeR and Linear Discriminant Analysis (LDA) (*p* < 0.01). **(C)** LDA of PICRUSt-predicted KEGG functions of microbial communities in the placentas from lean and obese subjects identified using LEfSe. Significant differences between groups are indicated as follows: ^∗∗^*p* < 0.01.

We next investigated if pregravid obesity alters the abundance and/or diversity of microbial communities within the placenta. Our analysis of 16S rRNA gene amplicon sequences revealed limited changes in microbial abundance and diversity in placenta of obese mothers. At the phyla level, a small expansion in relative abundance of Proteobacteria that was accompanied by a decrease in the abundances of Firmicutes and Bacteroidetes were noted (**Figure 5A**). However, only the relative abundance of the genus Bacteroides was significantly higher in placenta of obese mothers (**Figure 5B**).

To infer biological significance of these changes, we imputed metagenomic information from the microbial OTUs using PICRUSt. Linear Discriminant Analysis of these pathways using LEfSe identified 51 bacterial gene families predicted to be differentially expressed between placentas of lean and obese mothers (**Figure 5C**). Specifically, bacterial genes in pathways of amino acid metabolism and biosynthesis of unsaturated fatty acids were predicted to be enriched in placentas from obese mothers whereas families of genes involved in genetic information processing were predicted to be less abundant (**Figure 5C**).

## Discussion

The superimposition of low-grade systemic inflammation associated with obesity with the inflammatory state established during pregnancy is believed to impart risks for both the mother and the offspring. High pregravid BMI is associated with increased risks of postpartum infection (Cnattingius et al., 2013), premature delivery (Young et al., 2016) and preeclampsia (Rinaudo and Wang, 2012). Moreover, the suboptimal intrauterine environment alters the perinatal fetal programming, resulting in the development of metabolic syndrome (Thornburg and Marshall, 2015) (obesity, cardiovascular disease, and diabetes) and dysregulation of the immune system in the offspring (Segovia et al., 2017; Sureshchandra et al., 2017). Our understanding of the crosstalk between immune changes in the periphery and placenta dysfunction remains limited because studies to date have characterized the consequences of pregravid obesity either on systemic or placental inflammation. Therefore, in this study, we assessed the impact of pregravid obesity on systemic inflammatory and metabolic factors, frequency and responses of peripheral immune cells as well as the placental transcriptome and microbiome at 37 weeks of gestation.

As previously reported, pregravid obesity resulted in increased plasma levels of metabolic hormones such as insulin, leptin, and lipocalin-2, indicative of insulin resistance, mild gestational hyperglycemia, and metabolic syndrome (Bo et al., 2004). In line with previous studies, plasma levels of CRP and IL-6 were elevated while those of TNFα were comparable between the two groups (Christian and Porter, 2014). IL-6 has been shown to bi-directionally transfer across the placenta (Zaretsky et al., 2004), and indeed, we reported increased levels of IL-6 in the cord blood of babies born to obese mothers described in this cohort (Wilson et al., 2015). Transfer of maternal IL-6 into fetal circulation could result in low-grade inflammation in the offspring, altering development and maturation of the immune system. In support of this hypothesis, cord blood monocytes from babies born to the obese mothers in this cohort are hyporesponsive to LPS (Sureshchandra et al., 2017). Higher maternal plasma levels of IL-6 and CRP have also been associated with alterations in fetal growth and brain development (Sanders et al., 2014), wheezing and lower respiratory tract infections (Morales et al., 2011), the incidence of which is increased in offspring of obese mothers.

In addition to elevated levels of adipokines and pro-inflammatory cytokines, we observed increased plasma levels of innate cell chemo-attractants CCL22 and IL-8 as described for non-gravid obesity (Kim et al., 2006; Safa et al., 2016). Although frequencies of circulating DC, NK cells and monocytes were not altered, it is possible that their trafficking into tissues could be different, which in turn would impact the pathophysiology of pregravid pregnancy. For instance, increased recruitment of neutrophils to or alteration in balances of NK and DCs in the myometrium contributes to the pathophysiology of preterm labor (Gomez-Lopez et al., 2014). Plasma levels of the monocyte chemoattractant CCL4 on the other hand were reduced, which potentially could increase susceptibility to infection. We also report elevated levels of growth factors FGF2, EGF, and GM-CSF, which have been shown to impact placental development and fetal growth (Forbes and Westwood, 2010).

In addition to plasma levels of immune mediators, we investigated the impact of pregravid BMI and frequency/function of circulating maternal immune cells during third trimester. We detected no significant correlations between pregravid BMI and numbers of circulating WBCs, lymphocytes or granulocytes. However, we observed a decrease in naïve and concomitant increase in central memory CD4+ T cells in the obese group. Moreover, fewer CD4 T cells produced IL-17 while a higher frequency of CD4 T cells produced IL-4 following stimulation, suggesting a skewing towards Th2 and away from Th17 responses, as reported in obese individuals (van der Weerd et al., 2012). Decreased ability to produce IL-17 could lead to reduced protection against bacterial, fungal, parasitic, and viral infections, especially at mucosal surfaces (Ishigame et al., 2009; Sen et al., 2013). These findings are in line with clinical observations of increased incidence of postpartum genital tract infections, urinary tract infections, and wound infection in obese women (Sebire et al., 2001; Usha Kiran et al., 2005). Our findings, however, differ from data obtained during the second trimester where frequencies of CD4+ and CD8+ T-cells were reduced together with their ability to produce TNFα and INFγ (Sen et al., 2013). This discrepancy suggests that circulating lymphocyte populations undergo dynamic changes throughout pregnancy, a question requiring more in depth longitudinal studies.

Both obesity (Poitou et al., 2011) and pregnancy (Luppi et al., 2002) have been independently shown to be associated with increased frequencies of non-classical monocytes. In contrast, we report comparable frequencies of classical and non-classical monocyte population between the two groups. Stimulation with TLR2 and 4 ligands, resulted in greater production of pro-inflammatory mediators, potentially due to obesity-induced changes in TLR activation in myeloid cells (Ahmad et al., 2012). Furthermore, animal models of obesity have established that fatty acid mediated alterations in TLR2 and TLR4 signaling play a central role in the establishment of insulin and leptin resistance (Konner and Bruning, 2011).

Although number of circulating DCs was comparable, pDCs were significantly reduced in the obese group. Interestingly, circulating GM-CSF, a growth factor known to disrupt the balance between cDCs and pDCs during pregnancy (Fang et al., 2016) significantly increased with pregravid BMI. Despite a lower number of pDCs, we observed significantly higher levels of pro-inflammatory cytokine production in response to TLR3, TL7, and TL9 agonists, suggesting a substantial increase on a per cell basis. Recent studies have shown that aberrant nucleic acid sensing in peripheral tissue resident pDCs contributes to metabolic disease and insulin resistance in obese subjects (Revelo et al., 2016; Hannibal et al., 2017). In contrast to the increased secretion of cytokines and growth factors following TLR stimulation, production of several chemokines was reduced with pregravid obesity. This, in combination with aberrant inflammatory responses could result in increased severity of microbial infections. Indeed, there is a very strong association between pregravid maternal obesity and severity of postpartum wound, urinary tract and genital tract infections (Sebire et al., 2001; Paiva et al., 2012).

We next asked if pregravid obesity impacts the transcriptional profile of the placenta. The most variable placental genes that distinguish pregravid BMI play a role in oxygen transport, in line with reported alterations in maternal blood oxidant balance with pregravid obesity (Sen et al., 2014). These gene expression changes potentially signal adaptation to inflammation and oxidative stress. Response to oxidative stress is also evident from up-regulation of mitochondria encoded tRNA genes, which modulate codon usage of stress response genes and can participate directly as signaling molecules for stress responses (Kirchner and Ignatova, 2015). Genes involved in vitamin A metabolism, an important process in embryonic development and maturation of the immune system (Azais-Braesco and Pascal, 2000), were also down-regulated. Our list of DEGs overlapped significantly with that reported recently (Altmae et al., 2017), including suppression of genes that play a role in immunity (*IL1R2*, *IL2RB*, *TNFSF10*, and *FN1)* and pregnancy complications associated with pregravid obesity (e.g., intrauterine growth restriction, placental insufficiency, large for gestational age, miscarriage, and preeclampsia) (*IGFBP1*, *IGFBP2*, *PRL*, *TAC3*, *RBP4*, *DKK1*, *TIMP3*, *FSTL3*, and *IGFBP1*) (Okamoto et al., 2006; Zhang et al., 2013; Sober et al., 2015).

Because alterations in placental microbiome have been recently linked to spontaneous preterm birth (Prince et al., 2016) and gestational diabetes (Bassols et al., 2016), we investigated the impact of pregravid obesity on the placenta microbiome. The only notable difference we detected was an increase in Bacteroides species, which has been previously shown to increase in the gut microbiome of obese mothers during the third trimester (Collado et al., 2008). Moreover, babies born to obese mothers also have increased abundance of Bacteroides compared to babies born to lean mothers (Mueller et al., 2016) suggesting a vertical transfer of these bacterial communities. Despite the lack of large changes at the phyla level, additional in silico analyses predict significant alterations in amino acid and fatty acid metabolic pathways in the microbial community of placentas from obese compared to lean subjects.

In summary, data presented in this study indicate an establishment of a pro-inflammatory environment, both systemically and in the placenta, which in turn can alter maternal metabolic status and immune responses to infections. Additionally, a pro-inflammatory environment can alter programming of fetal development, increasing the risk of the offspring to develop metabolic disease, immune dysregulation, cognitive impairment, and cardiovascular disease. However, the exact mechanisms by which maternal factors alter developmental programming of the offspring are yet to be determined. Future research will have to focus on the immunological basis of this dysregulation within the placenta, understanding if transfer of nutrients across the placenta results in modulation of immune responses in the offspring. Understanding these processes will help identify mechanisms that can be directly targeted to reverse the detrimental effects of pregravid maternal obesity on fetal development. Another limitation of the current study is that our study population was overwhelmingly Caucasian. Future studies will seek to extend these analyses to include subjects from racial and ethnic minorities.

## Ethics Statement

This study was carried out in accordance with the recommendations of Institutional Ethics Review Boards of Oregon Health and Science University and the University of California, Irvine and approved by the same. All subjects gave written informed consent in accordance with the Declaration of Helsinki.

## Author Contributions

SS, NM, JP, KT, and IM conceived and designed the experiments. SS, RW, TB, and MR performed the experiments. SS and TB analyzed the data. SS and IM wrote the paper with input from NM, JP, and KT.

## Conflict of Interest Statement

The authors declare that the research was conducted in the absence of any commercial or financial relationships that could be construed as a potential conflict of interest.

## Footnotes

1 https://david.ncifcrf.gov/home.jsp
