# Supplementary figures and images for: Inflammatory Determinants of Pregravid Obesity in Placenta and Peripheral Blood
Source: Front Physiol. 2018 Aug 7;9:1089. doi: 10.3389/fphys.2018.01089 (PMC6090296; doi:10.3389/fphys.2018.01089)

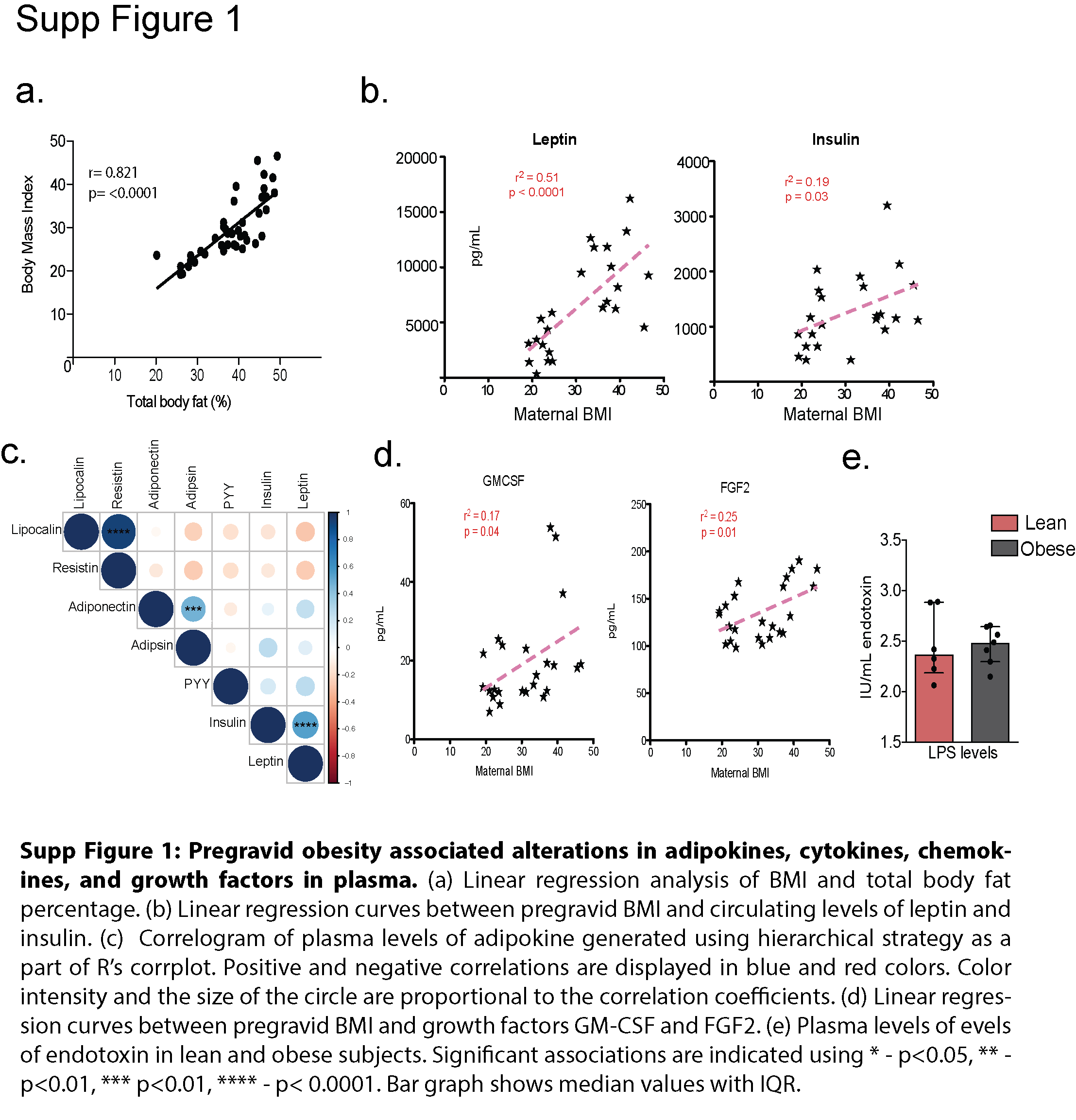

Supplement: Supplementary file 1 [file Image_1.TIF]

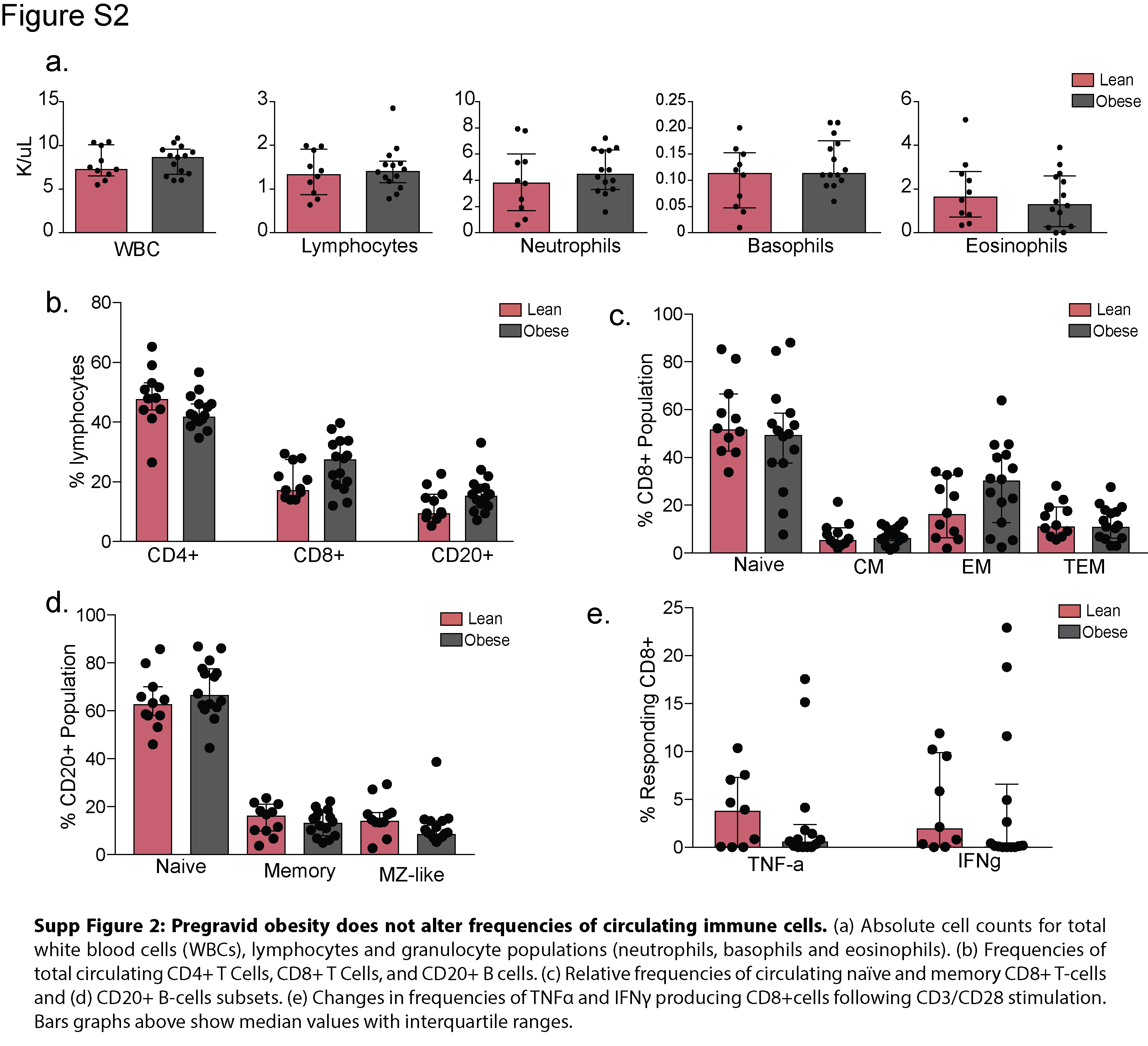

Supplement: Supplementary file 2 [file Image_2.TIF]

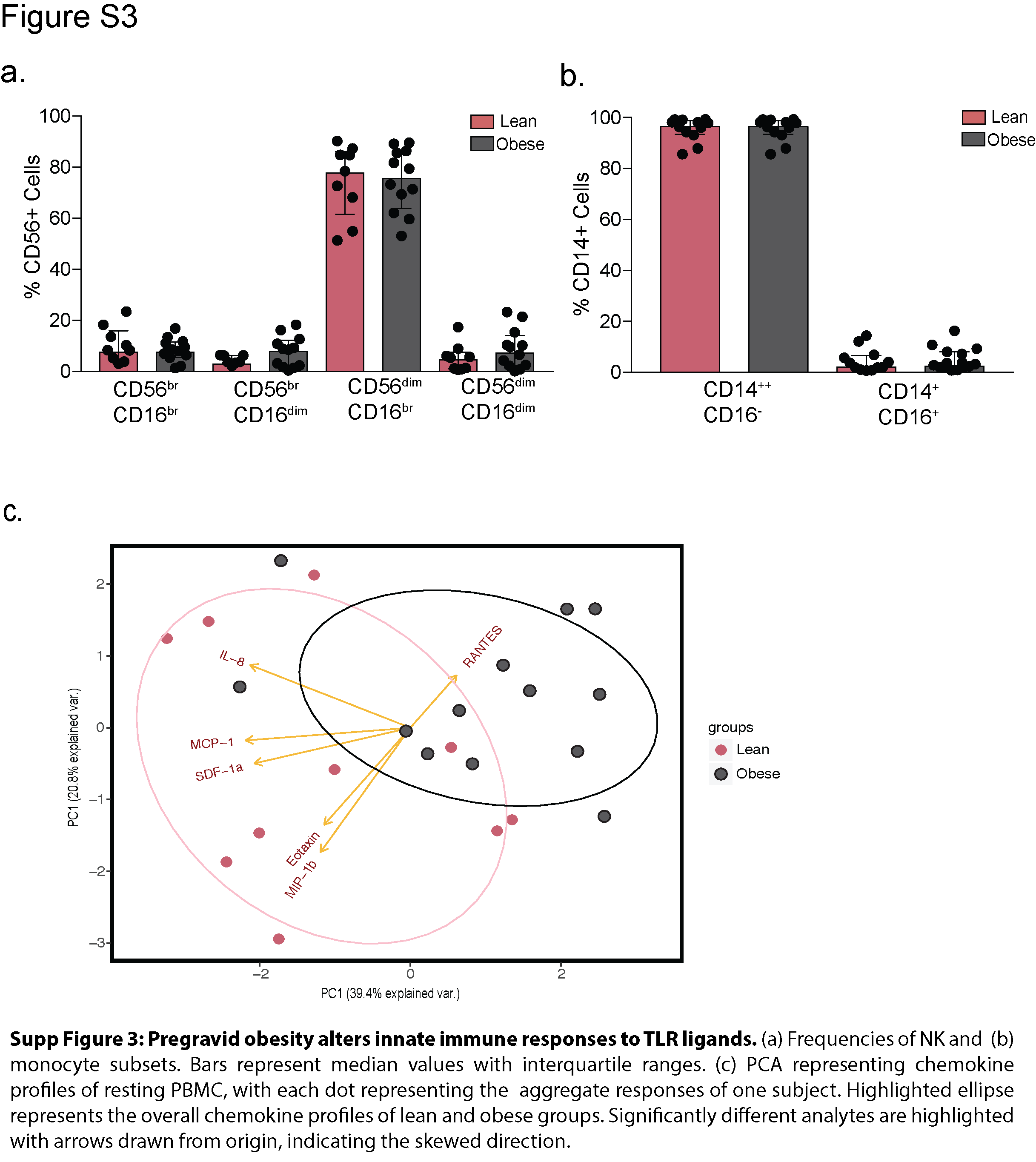

Supplement: Supplementary file 3 [file Image_3.TIF]

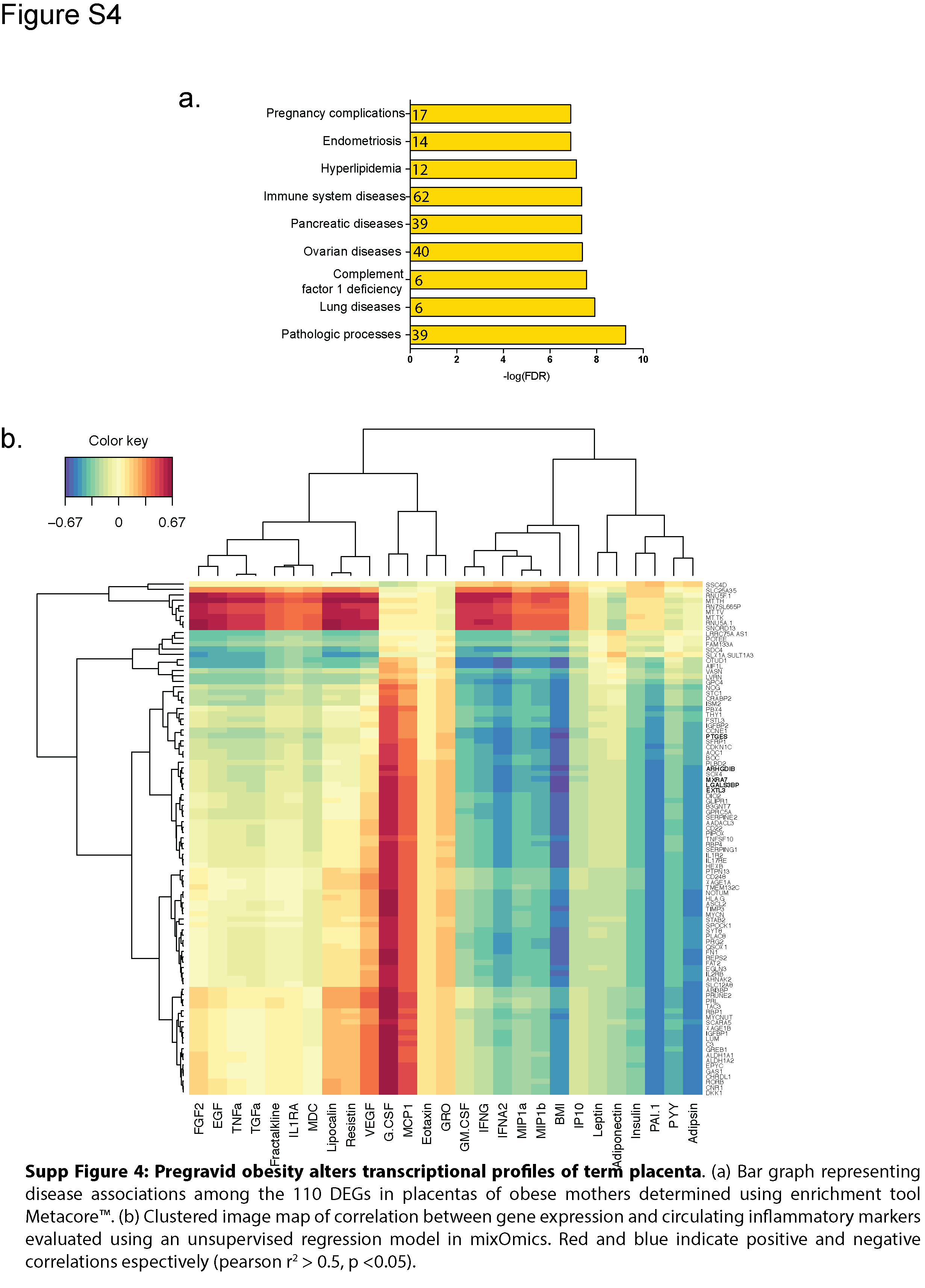

Supplement: Supplementary file 4 [file Image_4.TIF]
